# Research and Progress on Organic Semiconductor Power Devices

**DOI:** 10.3390/ma17133362

**Published:** 2024-07-08

**Authors:** Fangyi Li, Jiayi Zhou, Jun Zhang, Jiang Zhao

**Affiliations:** 1 School of Internet of Things, Nanjing University of Posts and Telecommunications, Nanjing 210023, China; b22100103@njupt.edu.cn; 2 Shanghai Qingwei Intelligent Technology Co., Ltd., Shanghai 200062, China; zzjjyy98@gmail.com; 3 College of Integrated Circuit Science and Engineering, Nanjing University of Posts and Telecommunications, Nanjing 210023, China; zhangjun1991@njupt.edu.cn

**Keywords:** organic, semiconductor, power devices

## Abstract

Organic semiconductor power devices have been attracting increasing attention due to their advantages such as flexibility, low fabrication cost, and sustainability. They have found wide applications in fields such as flexible electronic devices and biomedical devices. However, in the field of power applications, the lack of reliable organic semiconductor power devices is mainly attributed to the limited thermal stability and electrical stability of organic materials. This article provides a detailed review of the development status of organic semiconductor power devices from three aspects: device structure, organic materials, and fabrication methods. It clarifies that the future development goal is to enhance the voltage resistance and thermal stability of organic transistors through higher-performance structure design, higher-mobility materials, and higher-quality fabrication methods. The continuous innovation and development of the structures, materials, and fabrication of these devices will generate more novel devices, offering more possibilities for the application of organic semiconductor power devices. This information is of great reference value and guidance significance for engineers in related fields.

## 1. Introduction

As semiconductor power devices become more widespread in military applications, microwave communications, wearable devices, and medical equipment, there is a growing demand for power devices that offer transparency, can form large-area films, and are flexible. However, traditional inorganic semiconductor power device fabrication processes are complex, expensive, and have high hardness, making them difficult to bend, which no longer meets people’s needs. Organic semiconductor power devices [1,2], with advantages such as molecular-level structure control, flexibility [3] and plasticity, low fabrication cost [4], diverse performance capabilities, and sustainability [5,6], are attracting increasing attention from researchers and the industry [7,8,9,10]. Taehyun Park et al. consider the down-scalability of organic electronic devices. Their review comprehensively discusses the efforts and strategies aimed at overcoming the limitations of downscaling challenges in organic semiconductors [11]. In 2020, In Wang C. et al.’s review article, a concise retrospective of the development of organic field-effect transistors (OFETs) over the past few decades is presented in a chronological manner. The main focus is on the opportunities and challenges arising from advances in device physics, multifunctional materials, and integrated applications [12]. This is shown in Table 1.

In the past five years, organic semiconductor materials have emerged as promising new semiconductor materials in the field of microelectronics science and engineering. Achievements in the design, synthesis, and modification of novel organic molecules have improved their optoelectronic performance and stability. Research progress on devices such as organic field-effect transistors (OFETs) [18,19] and organic thin-film transistors (OTFTs) has been rapid, with significant breakthroughs in efficiency and longevity [20]. The composite application of organic semiconductors with other materials, such as optimizing organic–metal interfaces and combining organic semiconductors materials with inorganic materials, has provided new strategies for enhancing device performance.

However, organic semiconductor materials have lower charge carrier mobility, resulting in slower electron transport speeds, which limits their application in high-performance electronic devices. Furthermore, the stability and durability of organic semiconductor materials are relatively poor, making them susceptible to external environmental factors such as light and heat, leading to shorter device lifespans. OFETs with stable pressure and temperature resistance have not yet been widely applied, limiting the further development of organic-integrated circuits. To create OFETs with high temperature and high pressure resistance, improvements are needed in organic semiconductor power device structure, semiconductor materials, fabrication processes, and other aspects.

## 2. The Structure of Organic Semiconductor Power Devices

In the past decade, the field of organic semiconductor power devices has witnessed significant advancements, leading to the emergence of various device structures, each with unique working mechanisms and potential applications. Based on the positions of the gate electrode and organic semiconductor layer, as well as the contact positions of the organic semiconductor layer with the source and drain metal electrodes, there are four common structures of OFETs [21]: top-gate top-contact (TGTC), top-gate bottom-contact (TGBC), bottom-gate top-contact (BGTC), and bottom-gate bottom-contact (BGBC).

### 2.1. Top-Gate Device Structure

The device structure in which the gate electrode is located above the semiconductor layer is collectively referred to as a top-gate structure [22]. Top-gate structures are relatively more complex in processing compared to bottom-gate structures. This is because the gate electrode and source-drain electrodes require two depositions, in addition to the preparation of the gate dielectric layer and organic semiconductor layer. Top-gate structures also offer unique advantages. The gate dielectric layer in a top-gate structure can serve as an encapsulation, eliminating the need for an additional encapsulation layer.

**Figure 1:**
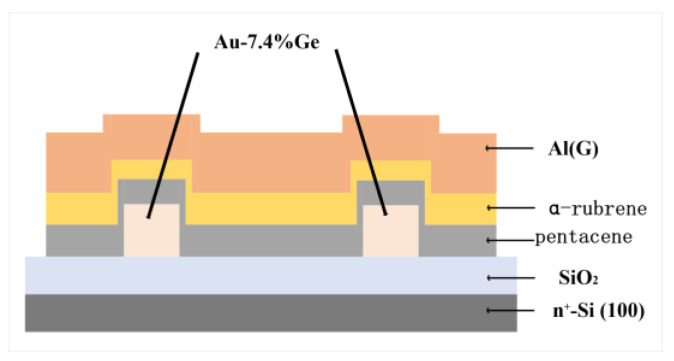
Cross-sectional view of top-gate top-contact organic field-effect transistor.

In 2010, M. Jagadesh Kumar proposed a top-gate-top-contact structure [23]. In 2018, Hiroki M. et al. proposed a top-gate-top-contact OFET [24]. This structure can be seen in Figure 1. The device utilizes α-amorphous rubrene as the gate insulating layer and achieves a top-gate structure through a delamination process. Research has demonstrated that α-rubrene exhibits good insulation properties, leading to a charge carrier mobility of 2.5~10^2^ cm^2^V^−1^s^−1^. This development holds promise for achieving high-density integrated and scalable OFET devices.

**Figure 2:**
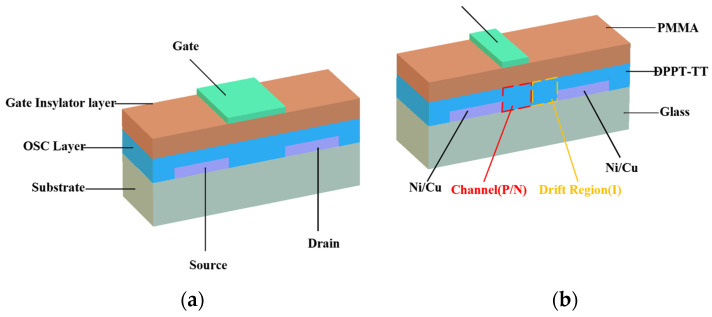
Cross-sectional view of top-gate bottom-contact organic field-effect transistor. (**a**) Traditional OFET, (**b**) LDR-OFET.

In 2022, Zhang Jun et al. proposed a lateral power device with a top-gate bottom-contact structure based on organic semiconductors [25], as shown in Figure 2. The aim is to achieve a high breakdown voltage. The device utilized Diketopyrrolidone pyrrolidine polymer and Polymethyl methacrylate (PMMA) as the semiconductor and insulating layer materials, respectively. By fabricating a lateral drift region organic field-effect transistor (LDR-OFET), the device demonstrated excellent blocking capability and on-state performance. With a drift region length of 20 μm, the device achieved a breakdown voltage of 2200 volts on a 50 nanometer-thick semiconductor layer deposited by spin-coating. The LDR-OFET, fabricated through a simple process at a low cost, offers a potential solution for high-breakdown voltage applications.

### 2.2. Bottom-Gate Device Structure

For bottom-gate structures, similar to traditional silicon-based devices, silicon wafer substrates can be used with silicon as the gate electrode and silicon dioxide as the gate dielectric layer. In this case, only the deposition of the organic semiconductor layer and the source-drain electrodes are needed, making the overall process simple. However, for flexible or transparent devices, silicon-based substrates are no longer suitable. In such cases, the bottom-gate structure no longer has the advantage of a simple process.

**Figure 3:**
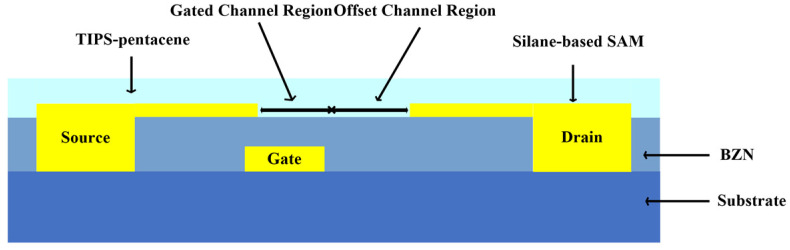
Cross-sectional view of bottom-gate top-contact organic field-effect transistor.

In 2018, Shih A. proposed a high-voltage organic thin-film transistor with a bottom-gate top-contact structure using a 6,13-bis(triisopropylsilylethynyl) pentacene solution treatment [26], as shown in Figure 3. By employing an offset channel architecture, the thickness of the organic semiconductor film could be reduced, thereby improving breakdown voltage, *I*_ON_/*I*_OFF_ current ratio, and output characteristics. The breakdown voltage of this device reached |*V*_DS_| > 450 V, which is three times higher than the previous results, offering high voltage tolerance and more flexible applications.

**Figure 4:**
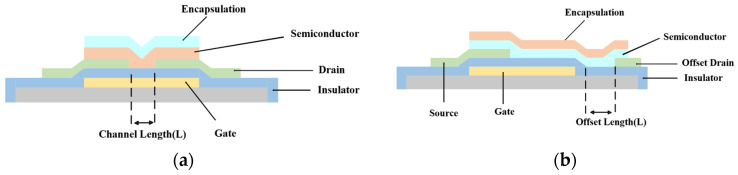
Cross-sectional view of top-gate bottom-contact organic field-effect transistor [13]. (**a**) OTFT (**b**) HVOTFT with drain offset.

In 2015, M.A. Smith et al. proposed a bottom-gate bottom-contact transistor [27], as shown in Figure 4. They fabricated a high-voltage organic transistor based on pentacene on a glass substrate. By setting an offset drain-source structure, the transistor could control a large drain-source voltage with a lower gate voltage, allowing the drain to withstand a high voltage of 300 V.

The above four types of structures were summarized and compared, as shown in Table 2.

In the structure of organic semiconductor devices, there is also a distinction between single-gate and dual-gate configurations. For single-gate OFETs, there is only one gate electrode which controls the flow of charge carriers in the channel. This design is simpler and easier to fabricate, but it may have limitations in terms of precise control over the device performance and characteristics.

On the other hand, dual-gate OFETs have two gate electrodes which allow for independent control of the channel conductivity and modulate the device behavior more effectively. This design offers improved performance in terms of device speed, stability, and sensitivity to external stimuli.

From the above structural summary, we can see that the current mainstream organic semiconductor devices OFET and OTFT have slightly better carrier mobility in OTFT than in OFET due to the differences in structure and materials.

Poornima Mittal et al. highlight the recent progress for organic small molecule and polymer-type OTFTs [36]. The review of Xiaojun Guo et al. focuses on constructing functional circuits and systems based on OTFTs. From various perspectives, it analyzes the performance specifications required for various circuit applications. Discussions are held on device structures and process choices for circuit integration in a manufacturable way [37].

Fuming Wu et al. reviewed the recent advances in the research of stretchable OFETs with high mobility, and the materials and healing mechanism of self-healing OFET are summarized in detail [20]. Xiaochen Ren et al. highlight recent advances in OFET-related energy topics, including low-power-consumption OFETs, NIR photodetectors, and organic thermoelectric devices [38].

However, many researchers are currently working on maximizing the mobility in OFETs based on their simple and low-cost fabrication process. Other comparisons between OFET and OTFT are shown in Table 3.

## 3. Materials for Organic Semiconductor Power Devices

Compared to traditional inorganic semiconductor materials, organic semiconductor materials have advantages such as mechanical flexibility, tunable optical bandgap, and low-temperature large-area film formation [40]. However, the breakdown voltage of current OFETs is much lower than that of traditional inorganic silicon-based semiconductor devices, making them unable to meet commercial demands [41]. Semiconductor professionals from various countries have been working for nearly thirty years to explore organic semiconductor materials that possess good thermal stability, good chemical stability, and high voltage resistance [42,43].

### 3.1. Polymers

Semiconductor polymers can be easily processed in solution to form high-quality films, have relatively high air stability, and can be printed on flexible substrates at low manufacturing costs for large-area electronic circuits. Therefore, semiconductor polymers are often used as channel materials in OFETs. In polymer molecules, charge carriers transfer along the polymer’s main chain between molecules, and the conjugation of the main chain depends mainly on the molecular structure and coplanarity. Therefore, research on polymer semiconductor materials mainly focuses on improving main chain conjugation and coplanarity.

In recent years, many high-performance polymer semiconductors with excellent properties have been developed [44,45,46,47]. For example, pentacene, rubrene, poly(3-hexylthiophene): P3HT, poly(3-octylthiophene): P3OT, and poly(3-alkylthiophene): P3AT.

**Figure 5:**
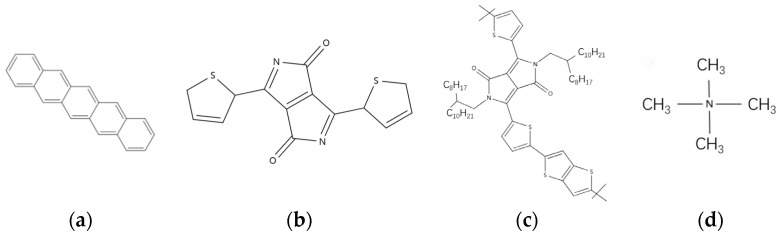
Molecular structure diagrams. (**a**) Pentacene (**b**) DPP-DPP molecule (**c**) DPPT-TT molecule (**d**) NMe4I molecule.

Pentacene is a preferable organic material due to its good optical transparency and good conducting properties [39]. It can be deposited at a low growth temperature to different substrates. The structure of pentacene is a polycyclic aromatic hydrocarbon having five benzene rings connected in a linear fashion as illustrated in Figure 5a.

Hiroki M. et al. fabricate OFETs with a top-gate configuration utilizing an α-rubrene gate insulator [24]. The 50 nm-thick α-rubrene gate insulator shows a low leakage current (2 × 10^−5^ A/cm^2^ at *V*_G_ = −1 V) and better mobility(2.5 × 10^−2^ cm^2^V^−1^s^−1^).

Ankit Verma et al. fabricated a fully solution-processed cheap organic TFT sensor for ammonia sensing utilizing the P3HT polymer as an active layer [48].

The conductivity of polymers can be controlled by doping or controlling the polymerization process [28]. Key factors include main chain planarity, energy levels, crystallinity, and film morphology. High-performance polymers include P-type, ambipolar, and N-type polymers [13,49], but lower electron mobility and poorer device stability hinder the application of ambipolar or N-type polymer FETs. Compared to N-type polymers, P-type polymer materials exhibit superior electrical performance.

The most widely researched P-type polymer at present is diketopyrrolopyrrole (DPP) and its derivatives. As shown in Figure 5a, the molecule of thieno[3,4-b]thiophene-diketopyrrolopyrrole is a derivative of DPP.

In 2012, C. Kanimozh et al. reported a novel conjugated copolymer based on diketopyrrolopyrrole-diketopyrrolopyrrole (DPP-DPP) [50], which can significantly improve electron mobility, with mobility values exceeding 3 cm^2^V^−1^s^−1^. Its extended absorption characteristics can be up to 1100 nm.

In 2013, Jung Ha Park et al. reported another DPP-based polymer that exhibited a high mobility of 2.36 cm^2^V^−1^s^−1^. Furthermore, after being stored in air for 7 months, the electrical properties of the polymer showed no significant change [51].

In 2013, Zhang Xinran et al. proposed that charge transport in high-mobility semiconducting polymers is quasi-one-dimensional. They provided new molecular design guidelines by examining an indacenodithiophene–benzothiadiazole copolymer having a field-effect mobility of up to 3.6 cm^2^V^−1^s^−1^ with a combination of diffraction and polarizing spectroscopic techniques [45].

In 2016, Hewei Luo et al. reported a new method to enhance the charge carrier mobility by inserting tetramethylammonium iodide into a DPP-conjugated polymer, as shown in Figure 5b. Without any additional treatment, the resulting thin film showed a hole mobility of up to 26 cm^2^V^−1^s^−1^ [52]. This is 24 times higher compared to the film without the ion additive under the same conditions (Figure 5c).

### 3.2. Organic Small Molecule Compound

Organic small molecule compounds used in organic semiconductor devices typically have the characteristics of simple structure, relatively low molecular weight, and a simple synthesis process. Chemical modifications can improve the stability and charge mobility of small molecules by substituting specific functional groups or ligands in the original molecules. This can be achieved by controlling their molecular structure, crystal morphology, and hybrid ligands. This enables excellent charge transport performance and controllable electrical properties. Common N-type organic small molecule semiconductor materials include halogenated small molecules, cyano-containing small molecules, quinone-like small molecules, etc. [53]. Common P-type organic small molecule semiconductors include perylene, phthalocyanine compounds, rubrene, etc. [54,55]. These materials can be used in the preparation of various devices such as organic field-effect transistors, organic photodiodes, organic light-emitting devices, and more.

In 2016, Feng L. et al. [56]. reported the fabrication of low-voltage OFETs on flexible substrates. The OFETs used poly(4-vinylphenol)(PVP) for the gates and dielectric, 6,13-bis(triisopropylsilylethynyl)-pentacene (TIPS-pentacene) blended with polystyrene (PS) as the semiconducting layer. The resulting transistor exhibited an operating voltage as low as 3 V, a mobility of 0.26 cm^2^V^−1^s^−1^, and a threshold voltage of 0.17 V.

In 2011, Chen et al. proposed that introducing pyridine is an effective way to obtain excellent N-type OFET materials [57]. By incorporating the pyridine ring into molecules such as tetracene and pentacene, on one hand, it can enhance the stability and electron injection capability of these molecules in air. On the other hand, pyridine can promote molecular π-stacking, thereby imparting good electron transport properties to the molecules, with mobility even comparable to some amorphous silicon devices.

In 2011, Liang Z. et al. reported the introduction of pyridine into pentacene derivatives, resulting in a carrier mobility as high as 3.3 cm^2^V^−1^s^−1^ [58].

In 2018, Chu M. et al. refreshed the record for N-type organic devices using tetrachloro-substituted derivatives, achieving a carrier mobility as high as 27.8 cm^2^V^−1^s^−1^ [59].

Similarly, in 2015, Krupskaya Y. et al. achieved a carrier mobility as high as 25 cm^2^V^−1^s^−1^ [60] in organic devices using fluorinated tetra-CNQ-dimethane as the semiconductor layer. With the continuous development of organic electronics, chemical synthesis has become one of the most important methods for improving the performance of organic small molecule materials.

**Figure 6:**
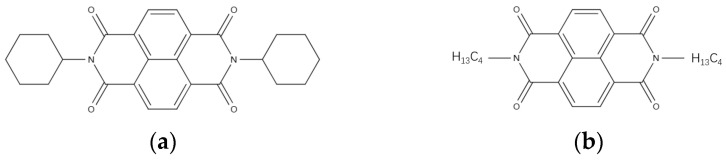
Schematic diagram of NDI molecule and its nitrogen substituent groups. (**a**) Molecular structure diagram of NDI molecule (**b**) Substitution of nitrogen substituent groups on NDI.

In 1996, D. Shukla et al. demonstrated that compounds formed by substituting alkyl or other groups at the nitrogen position of dicyanomethylene exhibited an electron mobility as high as 6.2 cm^2^V^−1^s^−1^ when measured in an argon environment, while the carrier mobility of naphthalene diimide (NDI) small molecules was only 10^−4^ cm^2^V^−1^s^−1^. This proves that functional group substitutions can enhance the carrier mobility of small molecules [61]. Functional group substitution has become an important method for improving the electron mobility of organic small molecule materials, as shown in Figure 6.

### 3.3. Factors Affecting Organic Materials

In addition to using different materials to make various organic semiconductor devices, the properties of organic materials can also be changed through doping or radiation methods.

1. Doping effects

Doping can not only change the type of organic matter [28], but also alter performance parameters such as conductivity and so on.

Yuliar Firdaus et al. [62] demonstrated that CuSCN could be doped with inorganic salts such as CuCl_2_ using a simple solution-processing route which could be extended in other solar cell technologies including perovskite and dye-sensitized solar cells.

Saurabh Pareek et al. [63] introduced graphitic carbon nitride, a two-dimensional graphene-like material, tuning electronic properties of PEDOT:PSS via secondary doping, thereby increasing the conductivity of doped PEDOT:PSS films by 75% and 30%, respectively, for the optimal doping concentrations. The polymer solar cell employing these films as hole transport layers yielded a power conversion efficiency of 7.65% and 6.44%, respectively, compared to 5.45% for the reference devices with pristine PEDOT:PSS as hole transport layers.

Pengchao Zhou et al. [64] provide that charge extraction efficiency can be improved by doping 2D material of antimonene quantum sheets into PEDOT:PSS emulsion. The interfacial properties between the hole extraction layer and PBDB-T:ITIC-based active layer were properly meliorated. Over 8.09% enhancement of power conversion efficiency is observed compared to that of the control cell with pure PEDOT:PSS.

Jie Luo et al. [65] fabricated the inverted polymer solar cells with pure and indium-doped ZnO as an electron transport layer. The result showed that the In-doped ZnO-based device had a high power conversion efficiency of 5.99%, and a nearly 40% improvement in comparison with the pure ZnO-based device without the UV treatment.

2. Irradiation effects

Irradiation can also greatly affect the properties of organic materials.

Takahiko Sasaki et al. [66] reported X-ray irradiation-induced carrier doping effects on the electrical conductivity of the organic dimer Mott-insulators *k*-(ET)_2_*X*. They observed a large decrease in the resistivity by 40% with the irradiation at 300 K and the metal-like temperature dependence down to about 50 K.

The behavior of polar metal organic molecules, particularly chloroaluminum phthalocyanine (ClAlPc), upon ultraviolet (UV) irradiation was investigated to evaluate the stability of the adsorption process on the Ag(111) thin film and bulk crystal [67]. ClAlPc in the Cl-down configuration was energetically more stable on the Ag thin-film surface than on the corresponding surface of the Ag bulk crystal.

Sourav Banerjee et al. [68] reported on a theoretical study focusing on the effect of charge transfer and chemical bonds on the dynamics of small biological molecules irradiated by a single X-ray-free electron laser pulse.

I. Karbovnyk et al. [69] obtained nanostructured organic thin films with polarized luminescence by means of the vapor deposition in the presence of polarized laser irradiation outside the absorption band. It was shown that irradiation of molecules by a non-resonant polarized laser beam during deposition strongly decreased the formation of aggregated molecules.

The organic semiconductor materials introduced above represent only a small fraction of the available materials. A comparison of the two types of materials is shown in Table 4. The performance of devices is also influenced by many factors such as molecular stacking, interfaces, thin film morphology, etc. All of these factors can be detrimental to the reproducibility of performance. With the continuous development of research technology, more high-performance organic semiconductor materials will emerge, expanding their applications in the field of microelectronics.

## 4. Preparation Methods of Organic Semiconductor Power Devices

In the field of microelectronics, the preparation methods of organic power semiconductors are one of the key research directions. Different preparation methods can affect the structure, morphology, and performance of organic power semiconductor materials. Most gate dielectrics in organic semiconductor power devices are based on polymers, making it difficult to deposit them using traditional thermal evaporation methods. However, the source and drain electrodes need to be in direct contact with the semiconductor layer. Therefore, the focus of research is on how to reduce contact resistance and improve carrier mobility without damaging the semiconductor layer.

### 4.1. Preparation by Solution Method

The solution method of preparation mainly involves dissolving organic semiconductor materials in organic solvents [45] and forming thin films on substrates through methods such as drop-casting and spin-coating. The advantage of drop-casting is that the process is very simple, but the disadvantage is that the organic semiconductor film may be uneven, with a higher proportion of disordered crystalline areas, leading to high electrical resistance.

In 2023, Ankit Verma et al. fabricated a fully solution-processed cheap OTFT sensor [48]. A simple, cost-efficient FTM method was used to coat a uniform film thickness of 25 ± 4 nm active polymer film on a solution-processed LaZrOx/HMDS film. At 5 ppm ammonia gas, the sensor had a high response of 47% and a short average response and recovery time of 9 and 50 s, respectively.

**Figure 7:**
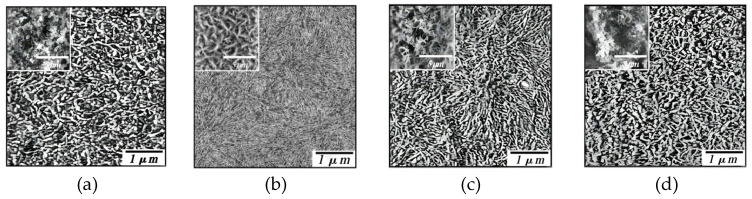
AFM images of P3HT films drop-cast on S_i_O_2_ substrate from different solvents [70]. (**a**) CH_2_Cl_2_, (**b**) toluene, (**c**) CHCl_3_, (**d**) THF. The insets represent AFM topography images with a larger scale.

In 2005, Hoichang Yang et al. [70] used the drop-casting method to dissolve organic semiconductor materials in different organic solvents and deposited the solution drops on a substrate (see Figure 7). After the evaporation of the organic solvent, organic semiconductor thin films were formed. The carrier mobility of the prepared thin-film transistor was in the range of 10^−4^~10^−2^ cm^2^V^−1^s^−1^.

The spin-coating method involves dropping a solution containing a dissolved organic semiconductor material onto a substrate, and then spinning it at high speed to form a uniform thin film across the entire substrate. Finally, the solvent is removed by heating annealing. Therefore, the spin rate and evaporation temperature are crucial as they directly control the thickness and uniformity of the film, thereby affecting the quality of the film. The advantages include simple process, low cost, capability for large-scale production, and preparation of flexible devices. However, the disadvantages are that organic films prepared by solution methods usually have low crystallinity and small grain size, leading to poor charge transport performance.

**Figure 8:**
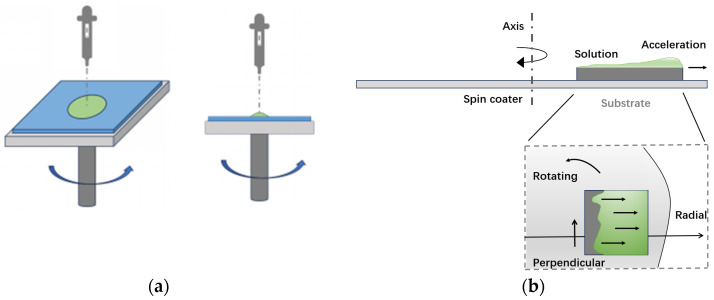
Schematic diagram of spin-coating process. (**a**) Dropping a solution containing dissolved organic semiconductor onto a substrate; (**b**) off-center spin coating process.

In 2014, Yuan Y. reported a highly aligned, simple off-center spin-coating method [71]. The schematic diagram of spin-coating process is shown in Figure 8. They used a thiophene-based polymer as the semiconductor, passivated the dielectric surface, and improved the continuity of the film. With this method, they achieved an ultra-high maximum hole mobility of 43 cm^2^V^−1^s^−1^.

### 4.2. Thermal Evaporation Method

The thermal evaporation method places organic semiconductor materials in a vacuum chamber and deposits them on the substrate surface through thermal evaporation or laser evaporation. Organic films prepared by this method have higher crystallinity and purity, with a more complete lattice structure, which is beneficial for improving charge carrier mobility and device performance. When the organic semiconductor material has higher crystallinity, it means that the molecules are more ordered and aligned in a regular pattern. This allows for the more efficient movement of charge carriers through the material, leading to higher mobility. Higher purity of the organic semiconductor material means that there are fewer impurities, defects, or contaminants that act as “traps” present in the material. The charge carrier mobility within the material can be enhanced, leading to better device performance, such as faster response times and higher current-carrying capabilities.

However, during the electrode deposition process, the high melting point of the metal leads to a high electrode deposition temperature, which can often cause thermal radiation damage to the organic semiconductor [19,72].

In 2002, Arndt C. Dürr et al. studied the thermal stability of the metal–organic interface under the thermal evaporation method [73]. They found that the interface properties are strongly determined by the preparation conditions of the gold film. Gold deposition at a high rate and low substrate temperature during the deposition process resulted in a clear interface with only a small amount of mutual diffusion. The substrate temperature could even be as high as 150 °C, which is sufficient to meet most technical applications of metal contacts on organic semiconductors. This is particularly important for addressing the thermal stability issues of devices operated at high temperatures.

### 4.3. Inkjet Printing Method

The Inkjet Printing Method is a process that uses inkjet technology to directly print organic semiconductor materials onto a substrate to form organic thin films [74,75]. By accurately controlling the inkjet head to create patterns, its advantage lies in the ability to create patterns directly without the need for a mask, thus saving materials significantly. It has the characteristics of high material utilization, non-contact, low cost, and high quality. However, organic thin films prepared by the inkjet printing method typically have a larger grain size and lattice defects, resulting in lower charge mobility. The operating voltage of the device is typically in the tens of volts, which is far from meeting the requirements for the high-voltage operation of power devices. It is suitable for the preparation of low-cost, large-area devices.

**Figure 9:**
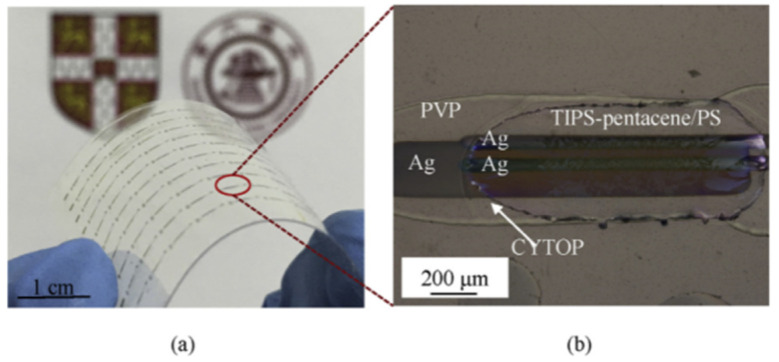
Flexible sample picture [56]. (**a**) Photograph of the prepared flexible sample. (**b**) Polarized optical micrograph of the sample.

In 2016, Feng L. et al. [56] mixed the crosslinking agent poly(melamine-co-formaldehyde) (PMF) with poly(4-vinylphenol) (PVP) in a 1:2 mass ratio, and dissolved them in a solution. The mixed solution was then used as the ink for the dielectric layer. All printing processes were completed using an inkjet printer (See Figure 9).

### 4.4. Self-Assembly Technology

Self-assembly technology is the use of organic molecules to self-assemble into specific structures of organic semiconductor materials, such as the source/drain electrodes of organic transistors. Self-assembly technology allows for precise control of the structure and arrangement of organic semiconductor materials, thereby improving device performance and stability.

**Figure 10:**
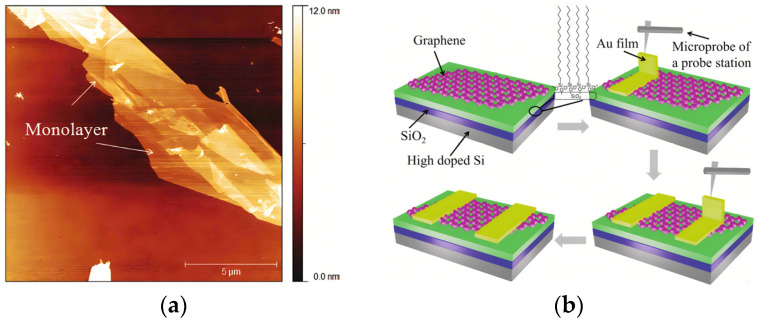
(**a**) AFM image of few-layer graphene on OTMS [76]; the scale bar is 5 μm. (**b**) Schematic illustration of the lithography-free process in GFET fabrication [76].

In 2011, Wang X. et al. successfully obtained high-performance graphene field-effect transistors (GFETs) by modifying SiO_2_/Si substrates using ordered self-assembled monolayers [76] (See Figure 10).

Therefore, a rational selection of processes and setting of process parameters will directly affect the device’s performance. It is necessary to optimize the fabrication process flow for organic semiconductor power devices. Please refer to the specific comparison in Table 5.

## 5. Summary and Outlook

Organic semiconductor power devices have been attracting much attention due to their advantages, such as molecular-level structure control, flexibility and adaptability, low fabrication cost, diverse performance characteristics, and sustainability. They have a wide range of applications in flexible electronic devices, new optoelectronic devices, biomedical devices, and other fields. This article provides a detailed overview of the development status of organic semiconductor power devices from the perspectives of device structure, organic materials, and processing methods.

In the past decade, organic semiconductor power devices have demonstrated a variety of device structures, each with unique working mechanisms and application prospects. Different device structures may lead to different device performances. The TC structure can improve the contact between the source-drain electrodes and the organic semiconductor layer. The BC structure allows the source and drain electrodes to be directly deposited on the substrate for easy patterning, effectively controlling the channel shape and achieving high-performance devices.

Organic semiconductor materials are a new type of material with excellent characteristics such as tunable bandgap, flexibility, and low cost. Significant progress has been made in the field of organic field-effect transistors based on donor–acceptor (D–A) polymers. Research on polymer semiconductor materials is mainly focused on improving their main chain conjugation and coplanarity. Several polymers have demonstrated carrier mobility exceeding that of amorphous silicon, meeting the requirements for flexible substrates. For practical applications, high-mobility and stable ambipolar or N-type OFET devices compatible with P-type devices need to be achieved.

Organic semiconductor materials have unique physicochemical properties, and the preparation methods of semiconductor layers are not limited to thermal evaporation deposition, but also include wet processes such as drop-casting, inkjet printing, and spin-coating. Printing technology has advantages such as high efficiency, material savings, low cost, and ease of large-area production, making it a promising manufacturing technique. However, future goals require further advancements in printing technology to achieve stable inks, high resolution, sub-micron patterning, and large-area film uniformity.

For many functional circuit applications, after the mobility reaches a certain level, material design for the overall performance such as sharp switching, low leakage current, and reliability becomes more important. It is recommended that the material and device development should take a whole set of application-oriented performance figures-of-merit instead of a single parameter as the optimization objective [37].

In summary, the continuous innovation and development of device structures, materials, and fabrication processes have provided more possibilities for the application of organic semiconductor power devices in various fields such as optoelectronic devices, biosensors, and flexible electronics. In the future, organic semiconductor power devices will continue to generate more novel devices, creating new opportunities and challenges for the field of microelectronics science and engineering.

## Figures and Tables

**Table 1:** Comparison between inorganic semiconductor power devices and organic semiconductor power devices.

	Inorganic Semiconductor Power Devices	Organic Semiconductor Power Devices	Advantages
Material Structure	Usually single-crystal or polycrystalline structure of inorganic crystal materials	Composed of organic molecules, molecular-level structure can be freely designed and controlled	Organic semiconductor material structure can be precisely controlled through chemical design, facilitating performance tuning and application customization
Preparation Process	Usually requires complex processes under high-temperature and high-vacuum conditions	Can be prepared using simple and low-cost methods such as solution processing	Organic semiconductor material preparation processes are simple, cost-effective, suitable for large-scale production and flexible device requirements
Optoelectronic Properties	Have high charge carrier mobility and conductivity	Exhibit broad emission characteristics and low charge carrier mobility	Organic semiconductor materials have unique advantages in flexible displays [13], lighting, etc.; inorganic semiconductor materials perform better in high power, high frequency electronic devices
Sustainability and Environmental Friendliness	Many are rare metals or high-energy materials with significant ecological impacts	Natural organic molecular composition, easy to degrade and recycle	Organic semiconductor materials have better environmental friendliness, meeting sustainable development requirements
Application Fields	Mainly used in high-power electronic devices, communications, etc.	Mainly used in flexible electronic devices, new optoelectronic devices, biomedical devices, etc. [14,15,16,17].	Organic semiconductor materials are suitable for flexible electronics and biomedical fields, while inorganic semiconductors materials have advantages in high power, high frequency, etc.

**Table 2:** Comparison of four types of organic power semiconductor structures.

	TGTC [23,24]	TGBC [25,28]	BGTC [26]	BGBC [27]
Structure Diagram	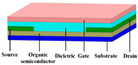	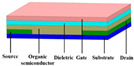	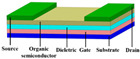	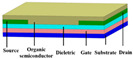
Gate and Organic Semiconductor Layer Position	Top Gate	Top Gate	Bottom Gate	Bottom Gate
Contact Position between Organic Semiconductor Layer and Source-Drain Electrodes	Top Contact	Bottom Contact	Top Contact	Bottom Contact
Process Complexity	High	High	Low [29]	Low
Packaging Requirement	No additional packaging layer required	No additional packaging layer required	Additional packaging required	Additional packaging required [30]
Suitability for Flexible or Transparent Devices	Suitable	Suitable	Not suitable for silicon-based substrates	Not suitable for silicon-based substrates
Substrate Deposition	Inconvenient for patterning	Inconvenient for patterning	Convenient for patterning	Convenient for patterning
Contact with Semiconductor Layer	Excellent	Excellent	Average	Average
Contact Resistance	No additional contact resistance	No additional contact resistance	Additional contact resistance incurred	Additional contact resistance incurred
Molecular Arrangement at Electrode-Channel Boundary	Molecular arrangement more advantageous [7,31]	Additional contact resistance, suppressing charge carrier mobility [32]	Relatively parallel	Additional contact resistance, suppressing charge carrier mobility
Photolithography Process	Immature	Immature	Mature	Mature
Advantages	Simple process, no additional packaging layer	No additional packaging layer, better contact	Simple process, gate dielectric layer acts as packaging	Simple process, source-drain electrodes can be patterned easily
Disadvantages	Complex process, packaging required	Complex process, additional contact resistance [33]	Additional packaging layer required, complex process [34,35]	Additional contact resistance, not suitable for flexible devices

**Table 3:** Comparison of OFET and OTFT.

	OFET [23,24,25]	OTFT [26,27,39]
mobility	lower	higher
	PS: Organic thin-film materials are used, which have lower crystallinity and orderliness.	PS: Organic single-crystal materials or highly ordered organic thin-film structures are used.
Electrode structure	Susceptible to contamination by metal impurities.	Insusceptible to contamination by metal impurities.
	PS: The organic semiconductor layer is directly in contact with the metal source and drain electrodes.	PS: The organic semiconductor layer is positioned on top of an organic insulating layer between the two metal electrodes,
Source-drain current	Higher source-drain current and power consumption	Lower source-drain current and power consumption
	PS: Direct contact between metal and organic semiconductor	PS: The organic insulating layer serves as an isolation layer between the organic semiconductor layer and the metal electrodes.
Fabrication process	Relatively simple and low cost	Relatively complex and high cost
	PS: The fabrication processcan be achieved on large-area substrates using simple methods such as solution casting.	PS: High-quality organic single crystals or highly ordered organic thin-film structures, resulting in complex fabrication processes, high fabrication costs and technical challenges.
Application field	Suitable for low-cost, large-area applications	Suitable for high-performance, low-power microelectronic devices.
	PS: Such as logic circuits, RF tags, and other fields.	PS: Such as flexible displays, sensors, and other fields.

**Table 4:** Comparison of two types of organic semiconductor materials.

	Polymer	Organic Small Molecule Compounds
Conductivity	High	High
Molecular Structure	Irregular and unordered, long-chain or branched structure	Regular and ordered, containing specific functional groups
Molecular Size	Large molecular weight	Small molecular weight
Physical Properties	Usually flexible and malleable	Usually have good crystallinity and solubility
Synthesis Method	Usually prepared through polymerization reactions	Usually prepared through organic synthesis chemical methods
High-Temperature Resistance	Normal	Normal
Research Focus	Improving main chain conjugation and coplanarity	Functional group substitution and chemical synthesis
Application Occasions	Flexible electronic devices, new optoelectronic devices, biomedical devices, etc.	Flexible electronic devices, new optoelectronic devices, biomedical devices, etc.

**Table 5:** Comparison of different preparation methods.

Preparation Method	Process Difficulty	Cost	Quality	Processing Time	Charge Transport Performance	Applicability	Factors Affecting
Solution Method	Low	Low	Moderate	Short	Poor	Large area, flexible	Spin-coating rate, concentration, annealing temperature
Vacuum Evaporation method	Medium	High	Excellent	Long	Excellent	High performance devices	Temperature, vacuum level, evaporation rate
Inkjet Printing method	Low	Low	Moderate	Short	Poor	Large area, low cost, flexible	Ink quality [77,78]
Self-assembly technique	High	Low	Excellent	Moderate	Excellent	Organic electronic devices	Surface treatment, solution concentration, temperature

## Data Availability

No new data were created or analyzed in this study.
